# Influence of Heavy Metals (Ni, Cu, and Zn) on Nitro-Oxidative Stress Responses, Proteome Regulation and Allergen Production in Basil (*Ocimum basilicum* L.) Plants

**DOI:** 10.3389/fpls.2018.00862

**Published:** 2018-07-05

**Authors:** Egli C. Georgiadou, Ewa Kowalska, Katarzyna Patla, Kamila Kulbat, Beata Smolińska, Joanna Leszczyńska, Vasileios Fotopoulos

**Affiliations:** ^1^Department of Agricultural Sciences, Biotechnology and Food Science, Cyprus University of Technology, Limassol, Cyprus; ^2^Institute of General Food Chemistry, Faculty of Biotechnology and Food Sciences, Lodz University of Technology, Łódź, Poland

**Keywords:** basil, reactive oxygen and nitrogen species, heavy metals, allergenic proteins, profilin

## Abstract

One of the most significant biosphere contamination problems worldwide is derived from heavy metals. Heavy metals can be highly reactive and toxic according to their oxidation levels. Their toxic effects are associated with the increased production of reactive oxygen species (ROS) and cellular damage induced in plants. The present study focuses on the effects of nickel (Ni), copper (Cu), and zinc (Zn) applied to the soil on the antioxidant response and allergen production in the aromatic plant basil (*Ocimum basilicum* L.) following a combined physiological, biochemical and analytical approach. The concentrations used for the three heavy metals were based on the 2002 Regulation of the Polish Ministry of the Environment on Soil Quality Standards [(i) agricultural land (group B): Ni 100 ppm, Ni 210 ppm, Cu 200 ppm, Cu 500 ppm, Zn 720 ppm and (ii) industrial land (group C): Ni 500 ppm, Cu 1000 ppm, Zn 1500 ppm, Zn 3000 ppm]. The highest physiological and cellular damage in basil plants was caused by Cu and Zn. Increasing concentrations of Cu resulted in a further increase in cellular damage and nitro-oxidative stress, correlating with an induction in activity of reactive oxygen and nitrogen species metabolism enzymes (SOD, CAT, APX, NR). Treatment with Cu led to increased concentration of the allergenic protein profilin, while increasing concentrations of Cu and Zn led to a decrease in the concentration of total proteins (likely due to proteolysis) and antioxidant capacity. Interestingly, severe Cu stress resulted in the accumulation of specific proteins related to transpiration and photosynthetic processes. On the basis of these findings, Ni stress in basil plants appears to be less damaging and with lower allergenic potential compared with Cu and Zn stress, while Cu-stressed basil plants experience most detrimental effects and display highest allergen production.

## Introduction

A large number of plant genera exists which belong to the Lamiaceae family and include common herb spices. One of them is the genus *Ocimum* which includes 50–150 species of herbs and shrubs (Javanmardi et al., 2003). Basil (*Ocimum basilicum* L.), a well-known herb, has been planted and used form ancient times and is abundant in tropical and subtropical regions (Khakdan et al., 2016). The leaves and flowers are used for medicinal and culinary purposes such as curing headaches, coughs, diarrhea and kidney malfunctions. In the extracts of these plants, a number of phenolic compounds (such as flavonoids, phenolic acids, and phenolic diterpenes) with strong antioxidant activity have been found. These antioxidant qualities have the ability to maintain health and prevent from coronary heart disease and cancer (Javanmardi et al., 2003).

A wide body of research focuses on the investigation of the effect of heavy metals on plant growth. Heavy metals are produced by anthropogenic pollution and are toxic and reactive. Their toxic effect is connected with the production of reactive oxygen species (ROS) and, as a consequence, an unbalanced cellular redox status (Pinto et al., 2003). The soil is contaminated by heavy metals from different sources such as industrial waste, agricultural fertilizers and roads (Teklić et al., 2008). This contamination affects the crops grown in the area. Heavy metals like cadmium (Cd), copper (Cu), nickel (Ni), lead (Pb), and zinc (Zn) may influence the condition of the plants in different ways. Some of them are vital elements for cellular metabolism (Cu, Zn, Ni), while some are non-essential (Cd, Pb) (Gratão et al., 2005). The increase of non-essential metals like Pb, Cd and micronutrients such as Zn, Cu and Ni may cause several negative aspects of oxidative stress (Nadgórska-Socha et al., 2013). If the effectiveness of a plant’s antioxidant defense is elucidated, it would therefore help to understand the tolerance defense mechanisms of plants to heavy metals.

Allergies are a common occurrence in modern societies. One approach to better understand the pathogenesis of allergic diseases is the characterization of allergens. For that reason, researches on plant extracts investigate the presence of allergens, one of which is profilin (Valenta et al., 1992). Profilins are 12–15 kDa monomeric actin-binding proteins that are found in the pollen of trees, grasses and weeds, and many fruits and vegetables (Assarehzadegan et al., 2010). They regulate the polymerization of actin and are present in all eukaryotic cells (Alvarado et al., 2014). Recent reports have shown that profilin could cause respiratory symptoms (Ruiz-Garcıa et al., 2011), whereas it is considered a mild food allergen producing local symptoms (Santos and Van Ree, 2011) such as oral allergy syndrome (OAS) because of its reduced enzymatic and thermal stability. Profilin food allergy therefore represents a secondary effect of common respiratory allergic diseases (Alvarado et al., 2014).

To our knowledge, this is the first report to quantify the impact of three heavy metals (Ni, Cu, Zn) applied to the soil on the antioxidant response and allergen production in the important aromatic plant basil compared with control plants following a multifactorial physiological, biochemical, and analytical approach.

## Materials and Methods

### Heavy Metals and Characteristics of Soil

Heavy metals applied to the soil were Ni, Cu, and Zn, each one in three concentrations. Metal concentrations were chosen based on the Regulation of the Polish Minister of the Environment on the Soil Quality Standards regarding soil and ground quality standards from 9^th^ September 2002. The lowest used concentrations correspond to the highest concentrations permitted in group B land or land classified as agricultural (except land under ponds and ditches), forest land and wooded shrubs, wasteland, as well as built-up and urbanized areas (with the exception of industrial, fossil and communication/trafficked lands), while the highest are the maximum allowable concentrations for a group C land or the industrial, fossil and communication/trafficked lands. The concentrations used for the three heavy metals were: [(i) group B: Ni 100 ppm, Ni 210 ppm, Cu 200 ppm, Cu 500 ppm, Zn 720 ppm and (ii) group C: Ni 500 ppm, Cu 1000 ppm, Zn 1500 ppm, Zn 3000 ppm]. The soil used in the experiment was a commercial soil from Gebr. Brill Substrate GmbH & Co. KG, with pH-value (CaCl_2_) = 5.8, 1.0 g/l salt content (KCl), 150 mg/l Nitrogen (N), 170 mg/l Phosphorus (P_2_O_5_) and 190 mg/l Potassium (K_2_O).

### Plant Material and Growth Conditions

A suitable dry weight of the soil mixed with perlite (ratio 3:1) (118 g per pot) was emptied onto a plastic foil, and was then combined with a solution of salts of heavy metals (NiCl_2_.6H_2_O, CuCl_2_ and ZnCl_2_) to obtain their respective ion concentrations (Ni 100, 210 and 500 ppm; Cu 200, 500, 1000 ppm; and Zn 720, 1500, 3000 ppm). Contaminated soil was left for 3 days (d), in order to bond the heavy metals with the matrix of the soil, and was subsequently properly hydrated.

**FIGURE 1:**
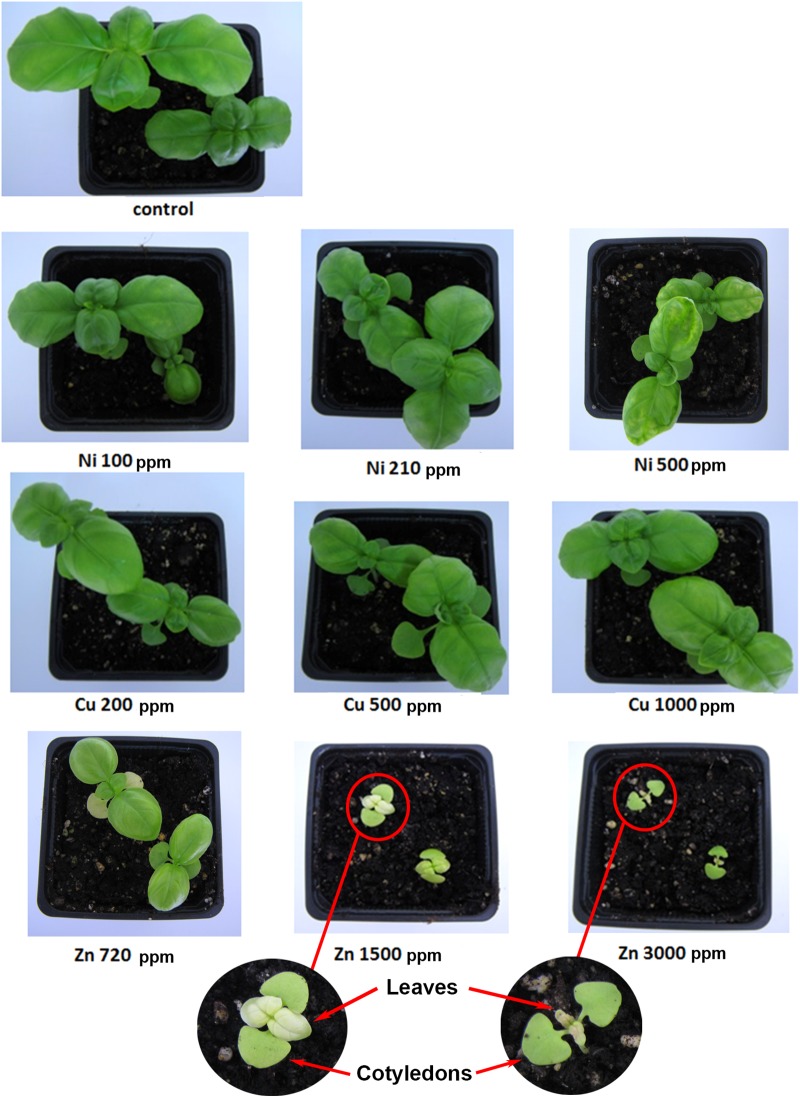
Aromatic basil plants treated with heavy metals (Ni, Cu, Zn) at three concentrations (including control plants). Phenotypic representation after transplantation of the plants at 33 days (harvest day).

Twenty five seeds of basil plants (Eden Company) per treatment were sown in the contaminated or control soil and placed at 4°C for 4 days for stratification. After incubation at low temperature, plants were transferred to a controlled environment growth room. Plants were grown at 25/20°C day/night temperature, at 40–70% humidity (RH) and a 16/8 h photoperiod. Detailed data for temperature (°C), Humidity (RH %) and light levels (Lux) in the growth room are shown in Supplementary Figure S1. After 17 days, germinated seedlings (Supplementary Figure S2) were transferred to smaller individual pots (2 seedlings/pot) with freshly prepared contaminated soil (62 g per pot) with heavy metals (as described above) in order to avoid potential competition for the light and compounds necessary for the development of plant. First and second leaves from 33 days old basil plants were used for all analyses (**Figure 1**). The harvested leaves were flash-frozen in liquid nitrogen, ground into fine powder using mortar and pestle, and stored at -80°C until use. The analyses were carried out using a minimum of three independent biological replicates (consisting of pooled samples from a minimum of three independent plants per replicate) in each treatment.

### Germination Rate (%)

Germination tests were conducted, each consisting of 25 seeds of basil plants placed in a pot containing wet soil. The first pot was considered to be a control (with no heavy metal treatment) and in the other pots, soil was contaminated each with the three heavy metals in three concentrations, similar to previous reports (Kaufmann and Ross, 1970; Woolley and Stoller, 1978; Bremner and Krogmeier, 1989; Singh et al., 2013). Number of seeds was counted to determine their germination rate. Germination rate is the percentage of the number of germinating seeds 17 days after planting over the total number of seeds sown (Supplementary Figure S2 and Supplementary Table S1).

### Photosynthetic Pigment Analysis

Hand-held Chlorophyll meter SPAD-502Plus (Konica Minolta Inc., Japan) was used. The measuring area was 2 mm × 3 mm, allowing small leaves to be measured (up to 1.2 mm thick). Measurements calculated an index in ‘SPAD’ units based on absorbance at 650 and 940 nm. Fourteen separate measurements with hand-held chlorophyll meter were made for each treatment.

For pigment extractions, 5 leaf disks (approximately 100 mg/ml) per sample were incubated in 5 ml DMSO for 30 min at 65°C. When the extractions were complete, samples were removed from the water bath and immediately spectrophotometric analysis was followed. Absorbance of samples was measured at 661, 643, 470, and 534 nm (TECAN, Infinite 200^®^ PRO) (Richardson et al., 2002). Chlorophylls (Chla, Chlb and total) and carotenoids concentrations were determined using the equations described by Misra and Dey (2013), while anthocyanins concentration was determined using the equations described by Nikiforou et al. (2014).

### Lipid Peroxidation Quantification

The extent of lipid peroxidation was determined from measurement of malondialdehyde (MDA) content resulting from the thiobarbituric acid (TBA) reaction (Filippou et al., 2011). The MDA content was measured at 532 and 600 nm (TECAN, Infinite 200^®^ PRO) and was estimated using the Lambert-Beer law, with extinction coefficient of 155 mM^-1^cm^-1^ and expressed as nmol MDA g^-1^ fresh weight (FW).

### Reactive Oxygen and Nitrogen Species Quantification

Hydrogen peroxide (H_2_O_2_) content was calculated spectrophotometrically based on the oxidation of iodide (I^-1^) to iodine (I), after the reaction of H_2_O_2_ with potassium iodide (KI), following the method of Loreto and Velikova (2001). The content of H_2_O_2_ was measured at 390 nm (TECAN, Infinite 200^®^ PRO) and was estimated based on a standard curve of known concentrations of H_2_O_2_ (μmol H_2_O_2_ g^-1^ FW).

Nitrite-derived nitric oxide (NO) content was determined using the Griess reagent in homogenates prepared in an ice-cold Na-acetate buffer (pH = 3.6) as described by Christou et al. (2013). NO content was measured at 540 nm (TECAN, Infinite 200^®^ PRO) by comparison to a standard curve of NaNO_2_ (nmol NO g^-1^ FW).

### Proline Content Quantification

The levels of free proline in leaf samples were measured using the ninhydrin reaction following the method proposed by Bates et al. (1973). Proline concentration was measured at 520 nm (TECAN, Infinite 200^®^ PRO) and was estimated using a standard curve and calculated on a fresh weight (FW) basis (μmol proline g^-1^ FW) (Filippou et al., 2011).

### Determination of Total Antioxidant Capacity

Two analytical techniques (ferric reducing/antioxidant power (FRAP) and phosphomolybdenum method) were employed to determine antioxidant capacity, using the same extracts for both. Antioxidants were extracted from the samples using the following procedure: One mL of methanol was added to 0.05 g of ground basil leaves and vortexed. Following, the mixtures were placed at 4°C for 48 h. Subsequently, samples were centrifuged for 10 min at 16000 × *g* at 4°C (Eppendorf Centrifuge 5415 R), and the supernatant was stored in sealed vials at -20°C for further analysis.

For the phosphomolybdenum method, 10 μL methanol extract was combined with 1 mL of reagent solution (0.6 mol L^-1^ sulfuric acid, 28 mmol L^-1^ sodium phosphate, and 4 mmol L^-1^ ammonium molybdate) and the tubes were incubated at 95°C for 90 min. The absorbance of the solution was measured at 695 nm (TECAN, Infinite 200^®^ PRO) against a blank sample, and the total antioxidant capacity was expressed as μmol ascorbic acid g^-1^ FW (Goulas et al., 2014).

For the FRAP method, a sample containing 1.98 mL of freshly prepared FRAP solution [0.3 mol L^-1^ acetate buffer (pH = 3.6) containing 10 mmol L^-1^ 2,4,6- tripyridyl-1,3,5-triazine (TPTZ) and 40 mmol L^-1^ FeCl_3_10H_2_O] and 15 μL of methanol extract were incubated at 37°C for 4 min and the absorbance was measured at 593 nm (TECAN, Infinite 200^®^ PRO). A standard curve of L-ascorbic acid was prepared and results were expressed as μmol ascorbic acid g^-1^ FW (Goulas et al., 2014).

### Enzymatic Activity Assays

#### p5CS

Plant cell extraction and p5CS activity measurements were processed according to Wang et al. (2011). Leaves (0.035 g) were homogenized in an extraction buffer (100 mmol L^-1^ Tris-Cl, pH = 7.5, 10 mmol L^-1^ β-mercaptoethanol, 10 mmol L^-1^ MgCl_2_, 1 mmol L^-1^ PMSF) using a mortar and pestle on ice. Extracts were centrifuged at 4°C for 20 min at 16000 × *g* (Eppendorf Centrifuge 5415 R). Supernatants were further clarified by centrifugation at 16000 × *g* (Eppendorf Centrifuge 5415 R) for 20 min at 4°C. p5CS enzymatic assay was carried out in 100 mmol L^-1^ Tris-Cl (pH = 7.2), 25 mmol L^-1^ MgCl_2_, 75 mmol L^-1^ Na-glutamate, 5 mmol L^-1^ ATP, 0.4 mmol L^-1^ NADPH, and the appropriate crude protein extract (100 μL). The reaction velocity was measured as the rate of consumption of NADPH, monitored as the decrease in absorption at 340 nm (TECAN, Infinite 200^®^ PRO) as a function of time. Total protein content was determined according to Bradford method (Bradford, 1976). p5CS specific enzyme activity was expressed as units mg^-1^ protein.

#### Nitrate Reductase (NR)

The assay was performed essentially as described (Liu et al., 2011), with some modifications. The buffer used for preparation of crude extracts (200 μL) contained 100 mmol L^-1^ potassium phosphate (pH = 7.5), 5 mmol L^-1^ (CH_3_COO)_2_Mg, 10% (v/v) glycerol, 10% (w/v) polyvinylpyrol-lidone, 0.1% (v/v) Triton X-100, 1 mmol L^-1^ EDTA, 1 mmol L^-1^ DTT, 1 mmol L^-1^ PMSF, 1 mmol L^-1^ benzamidine (prepared fresh) and 1 mmol L^-1^ 6-aminocaproicacid. Leaf tissue (0.025 g) was extracted in the appropriate buffer using a mortar and pestle and the mixture was thoroughly homogenized. Cell extract was centrifuged at 16000 × *g* (Eppendorf Centrifuge 5415 R) for 15 min and the clear supernatant was used immediately for measurement of enzyme activity (Wray and Filner, 1970). Total protein content was determined according to Bradford method (Bradford, 1976). NR activity was measured at 540 nm (TECAN, Infinite 200^®^ PRO) and was expressed as specific enzymatic activity (units mg^-1^ protein).

#### Antioxidant Enzymes

For superoxide dismutase (SOD) and catalase (CAT) extraction, leaf samples (0.05 g) were homogenized in ice-cold extraction buffer (100 mmol L^-1^ phosphate buffer pH = 7.5, 0.5 mmol L^-1^ EDTA, 1 mmol L^-1^ PMSF) using a mortar and pestle. Each homogenate was centrifuged at 16000 × *g* (Eppendorf Centrifuge 5415 R) at 4°C for 20 min and total supernatant was used for enzymatic activity assay. All steps in the preparation of extracts were carried out on ice. Total SOD activity was determined by measuring its ability to inhibit the photochemical reduction of nitro blue tetrazolium chloride (NBT) as described by Giannopolitis and Ries (1977), with minor modifications. The reaction mixture contained 50 mmol L^-1^ phosphate buffer (pH = 7.8), 13 mmol L^-1^ methionine, 75 μmol L^-1^ NBT, 0.1 mmol L^-1^ EDTA, 2 μmol L^-1^ riboflavin and 50 μL of enzyme extract in a final assay volume of 1.5 mL. The photoreduction of NBT was assayed spectrophotometrically at 560 nm (TECAN, Infinite 200^®^ PRO) and it was inversely proportional to SOD activity (Jiang and Zhang, 2002). The reaction mixture with no enzyme developed maximum color due to maximum reduction of NBT and was taken as control. The blank solution had the same complete reaction mixture but it was kept in the dark. One unit of SOD activity (U) was defined as the amount of enzyme required to cause 50% inhibition of the NBT photoreduction rate. The results were expressed as specific activity units mg^-1^ protein. Catalase (CAT) activity was measured according to Aebi (1984) with minor modifications. The reaction mixture consisted of 50 mmol L^-1^ potassium phosphate buffer (pH = 7), 10 mmol L^-1^ H_2_O_2_ and 200 μL enzyme extract to a final volume of 1.5 ml. The rate of H_2_O_2_ disappearance (extinction coefficient 39.4 mM^-1^ cm^-1^) was monitored at 240 nm (TECAN, Infinite 200^®^ PRO) during 1 min. The results were expressed as specific activity units mg^-1^ protein. Protein content of all samples was determined by the Bradford method (Bradford, 1976).

For ascorbate peroxidase (APX) extraction, leaf samples (0.05 g) were homogenized in ice-cold extraction buffer (50 mmol L^-1^ phosphate buffer pH = 7, 1 mmol L^-1^ EDTA, 1 mmol L^-1^ PMSF, 1% (w/v) PVP and 1 mmol L^-1^ ascorbic acid) using a mortar and pestle. All steps in the preparation of extracts were carried out on ice. Each homogenate was centrifuged at 16000 × *g* (Eppendorf Centrifuge 5415 R) at 4°C for 20 min and total supernatant was used for enzymatic activity assay. Ascorbate peroxidase (APX) activity was determined according to Jiang and Zhang (2002) with minor modifications. The reaction mixture consisted of 50 mmol L^-1^ potassium phosphate buffer (pH = 7), 0.5 mmol L^-1^ ascorbic acid, 0.1 mmol L^-1^ H_2_O_2_ and 15 μL enzyme extract to a final volume of 1 ml. H_2_O_2_-dependent oxidation of ascorbate was followed by a decrease in the absorbance at 290 nm (TECAN, Infinite 200^®^ PRO), using an extinction coefficient 2.8 mM^-1^ cm^-1^. The results were expressed as specific activity units mg^-1^ protein. Protein content of samples was determined by the Bradford method (Bradford, 1976).

### Protein Analyses

For performing protein content, SDS-PAGE and ELISA tests, fresh tissue was lyophilized using a Laboratory Freeze Dryer (Christ Alpha 1-4 LDplus), and then stored at room temperature. Basil samples were extracted with Tris-glycine buffer (T-G, pH = 8.3) composed of 0.05 M Tris and 0.33 M glycine. For this purpose, 0.05 g of dried plant tissue was homogenized in 3 ml of T-G buffer and incubated on a laboratory shaker (TTS 2, Yellow Line, IKA -Werke GmbH & Co. KG, Staufen, Germany) for 2 h. Samples were then centrifuged at 4000 rpm for 10 min and the supernatants were transferred into new tubes and stored until analysis at -20°C.

#### Determination of Protein

Protein concentration was determined by Pierce method using the Pierce^TM^ BCA Protein Assay Kit (Thermo Fisher, 23225) following the manufacturer’s instructions with bovine serum albumin (BSA) as a standard and expressed in mg g^-1^ dry weight (D.W.).

#### SDS-PAGE Analysis

Sodium dodecyl sulfate - polyacrylamide gel electrophoresis (SDS-PAGE) (15% separating gel, 5% stacking gel) was performed according to Laemmli (1970) in 10x Tris-glycine running buffer, pH = 8.3 (7.575 g Tris base, 36 g glycine, 2.5 g SDS in 250 ml H_2_O and diluted 10 times before use). Separation was conducted in Blue Vertical Mini Slab Gel System (BV102) (Serva, Heidelberg, Germany). Extracts were mixed with sample buffer (ratio: 1:1) which contained deionized water, 0.5 M Tris-HCl (pH = 6.8), glycerol, 10% (w/v) SDS, 0.5% (w/v) bromophenol blue and β-mercaptoethanol. Samples were denatured by heating to 95°C for 5 min and were then deposited on the gel (40μg protein per well). Pierce^TM^ unstained protein MW Marker (Thermo Fisher, 26610) was used as a molecular weight standard. Electrophoresis was performed at a constant voltage of 80 V (Desatronic 3000/200, Desaga, Heidelberg, Germany) for stacking gel (5%), increased to 130 V when samples underwent to separating gel (15%). Obtained electroforegrams were analyzed in Gelscan program.

#### LC-MS-MS/MS

Proteins were analyzed in Mass Spectrometry Laboratory, Institute of Biochemistry and Biophysics, Polish Academy of Sciences, Warsaw. In brief, peptide mixtures were analyzed by liquid chromatography coupled to tandem mass spectrometry (LC-MS-MS/MS) using Nano-Acquity (Waters) LC system and Orbitrap Velos mass spectrometer (Thermo Electron Corp., San Jose, CA, United States). Prior to the analysis, gel slices were subjected to standard “in-gel digestion” procedure during which proteins were reduced with 100 mM DTT (30 min at 56°C), alkylated with iodoacetamide (45 min in darkroom at room temperature) and digested overnight with trypsin (sequencing Grade Modified Trypsin - Promega V5111). Resulting peptides were eluted from the gel with 0.1% (v/v) trifluoroacetic acid (TFA), 2% (v/v) acetonitrile (ACN). Peptide mixture was applied to RP-18 precolumn (nanoACQUITY Symmetry^®^ C18 – Waters 186003514) using water containing 0.1% (v/v) TFA as mobile phase and then transferred to nano-HPLC RP-18 column (nanoACQUITY BEH C18 – Waters 186003545) using an acetonitrile gradient [0–35% (v/v) ACN in 70 min] in the presence of 0.05% (v/v) formic acid with the flow rate of 250 nl/min. Column outlet was directly coupled to the ion source of the spectrometer working in the regime of data dependent MS to MS/MS switch. A blank run ensuring lack of cross contamination from previous samples preceded each analysis.

Acquired raw data were processed by Mascot Distiller followed by Mascot Search (Matrix Science, London, United Kingdom) against Swiss-Prot database or NCBInr with restricted taxonomy to Green Plants. Search parameters for precursor and product ions mass tolerance were 10 ppm and 0.01 Da (after internal calibration by an in-house software), respectively, enzyme specificity: trypsin, missed cleavage sites allowed: 1, fixed modification of cysteine by carbamidomethylation and variable modification of asparagine and glutamine deamidation and methionine oxidation. Peptides with Mascot Score exceeding the threshold value corresponding to <1% False Positive Rate, calculated by Mascot procedure, and with the Mascot score above 30 were considered to be positively identified.

#### Profilin ELISA Analysis

Extracts were diluted 10 times in 0.1 mol/L carbonate/ bicarbonate buffer pH = 9.6 (Sigma-Aldrich, C3041). Hundred microliter of solution aliquots were used to coat Microtiter plates EB 92029330 (Labsystems, Helsinki, Finland) and incubated overnight at 4°C. Next, the plates were washed four times with PBS buffer and free binding sites were blocked by incubation with 3% (w/v) solution of skin milk in PBS (pH = 7.2), containing 0.1% (v/v) of Tween-20, for 2 h at room temperature. This was followed by removal of milk buffer solution, rinsing the plates four times with PBS buffer and further incubation with 100 μl of 1000-fold diluted anti-profilin antibodies [Anti-Profilin 1 (C-terminal) antibody produced in rabbit (Sigma-Aldrich, P7624)] for 2 h at room temperature. The plates were washed again and subjected to addition of 100 μl secondary antibody Anti-Rabbit IgG conjugated with alkaline phosphatase (Sigma-Aldrich, A3687) 5000-fold diluted. After incubation of the plates for 1 h and rinsing four times with PBS buffer, 100 μl of Alkaline Phosphatase Yellow (pNPP) Liquid Substrate (Sigma-Aldrich, P7998) was added and plates were incubated again for 30 min. Reaction was stopped by adding 100 μl of 3 M solution of NaOH and the output absorbance was measured using Multiscan RC reader at 405 nm. The immunoreactivity was normalized relative to the content of profilin from peach (Słowianek et al., 2016).

### Statistical Analysis

Statistical analysis was carried out using the software package SPSS v17.0 (SPSS Inc., Chicago, IL, United States) and the comparison of averages of each treatment was based on the analysis of variance (One-Way ANOVA) according to Duncan’s multiple range test at significance level 5% (*P* ≤ 0.05).

## Results

### Aromatic Basil Plants Treated With Various Concentrations of Heavy Metals (Ni, Cu, Zn)

Samples were analyzed using the ICP-MS method, which revealed that increasing concentrations of heavy metals (Ni 100–500 ppm, Cu 200–1000 ppm, and Zn 720 ppm) applied to treated plants correlates with increasing ion accumulation in plant leaves, thus validating the successful application of heavy metals as previously reported in Szczodrowska et al. (2016).

After 17 days, no phenotypic differences were observed in basil seedlings between Cu (200, 500, 1000 ppm), Zn 720 ppm and control plants, except for Ni (100, 210, 500 ppm), which showed lower germination rate compared with control plants, and the higher concentrations of Zn (1500, 3000 ppm) which appeared to have chlorotic leaves (Supplementary Figure S2).

As shown in **Figure 1**, macroscopic observation after 33 days revealed no phenotypic differences between Ni (100, 210 ppm) and control plants, whereas chlorotic leaves were observed in plants treated with Ni 500 ppm. Plants treated with Cu (200, 500, 1000 ppm) compared with control plants displayed similar phenotypes, as no district modulation in plant growth and development was observed. Macroscopic observations between plants treated with Zn 720 ppm and control plants showed that Zn 720 ppm resulted in smaller, chlorotic leaves. Moreover, Zn 1500 ppm in basil plants showed increased damage levels indicated by small, chlorotic leaves compared with control plants. The highest concentration of Zn 3000 ppm in plants compared with control plants appeared to be toxic. Some plants revealed very small chlorotic leaves, but most plants showed extensive necrosis in leaves before harvest day (33 days).

### Germination Rate (%) of Heavy Metal-Treated Basil Plants

A comparison was performed between the germination process of control plants (basil) and plants grown in contaminated soil with different concentrations of Ni, Cu, and Zn (Supplementary Table S1). In Ni-contaminated soil, the germination rate of Ni-treated seeds was lower than that in control plants, with increasing Ni concentrations however leading to increasing germination rates. In the case of Cu soil contamination, the germination rate for 200 ppm was higher that the germination rate of control plants, whereas higher Cu concentrations resulted in similar germination rates to that of control plants. Basil seeds sown in soil with Zn at 720 ppm showed a germination rate higher than that of control plants. As Zn concentration increased to 1500 ppm, the germination rate decreased at levels lower than that of control samples, but as Zn concentration increased further at 3000 ppm, germination rate was higher than that of control plants. It should be noted that even though the germination rate in this case was higher than in control samples, seedlings experienced severe chlorosis of the leaves (**Figure 1**).

### Effect of Heavy Metals on Physiological Parameters of Basil Plants

**FIGURE 2:**
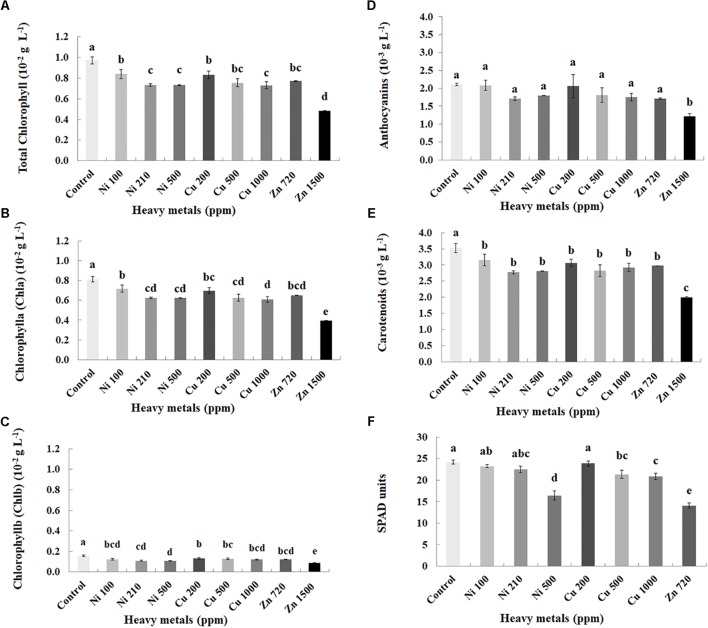
Effects of heavy metals (Ni, Cu, Zn) at three concentrations (including controls), on photosynthetic pigment content (**A** – total chlorophyll, **B** – chlorophyll a (Chla), **C** – chlorophyll b (Chlb), **D** – anthocyanins, **E** – carotenoids) and **F** – SPAD units of aromatic basil leaves (data are means ± SE of three replications for **(A–E)** and fourteen replications for **(F)**; SPAD of Zn 1500 ppm and Zn 3000 ppm-treated samples was not measured due to the small size of the leaves, while pigments were also not quantified for Zn 3000 ppm-treated samples due to lack of tissue). Bars with different letters are significantly different at *P* ≤ 0.05.

Plant pigments were determined in leaves of basil plants of three heavy metals (Ni, Cu, Zn) in three concentrations applied to the soil, compared with control plants (**Figures 2A–E**). Zn 3000 ppm-treated samples were not measured because of lack of sufficient tissue to perform this analysis (severely damaged). Chlorophylls (Chla, Chlb, and total), carotenoids and anthocyanins decreased with increasing concentration of heavy metals (Ni, Cu, and Zn) in contaminated soil, while content of all pigments was significantly lower in all heavy metal-treated plants compared with control ones.

Physiological parameters were further monitored through SPAD units measurements in leaves of basil plants of three heavy metals (Ni, Cu, Zn) in three concentrations applied to the soil, compared with control plants (**Figure 2F**). Zn 1500 ppm and Zn 3000 ppm-treated plants were not measured because it was not possible to perform the experiment due to the small size of the leaves. SPAD units decreased with increasing concentration of heavy metals (Ni and Cu) in contaminated soil. Photochemical efficiency of SPAD units was not significantly affected with Ni (100, 210 ppm) and Cu 200 ppm, whereas it was significantly lower in Ni 500 ppm, Cu (500, 1000 ppm) and Zn 720 ppm, compared with control plants.

### Cellular Damage in Heavy Metal-Treated Basil Plants Compared With Controls

**FIGURE 3:**
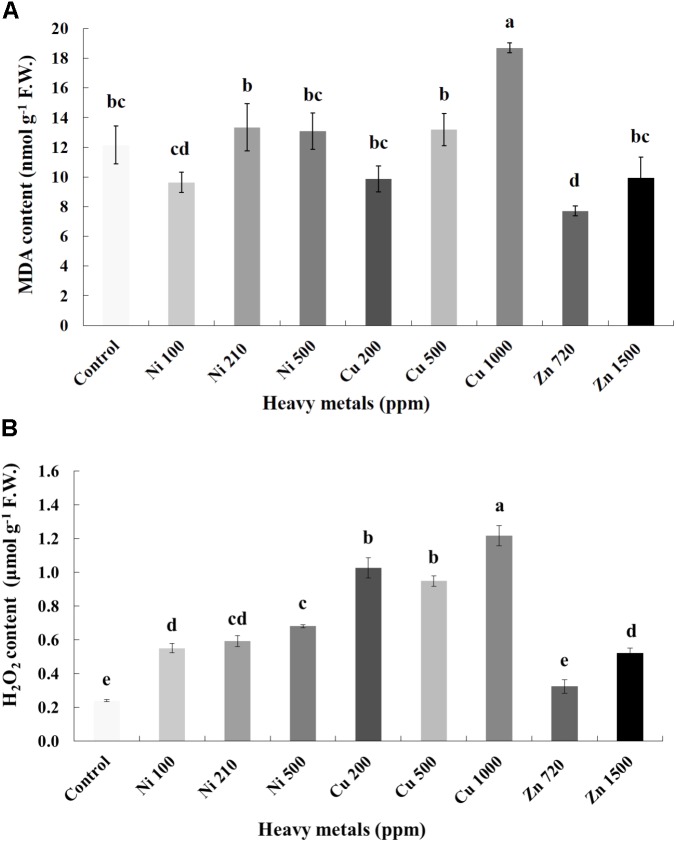
Effects of heavy metals (Ni, Cu, Zn) at three concentrations (including controls), on **(A)** malondialdehyde (MDA) content, and **(B)** hydrogen peroxide (H_2_O_2_) content of aromatic basil leaves. Data are means ± SE of three replications. Bars with different letters are significantly different at *P* ≤ 0.05. Zn 3000 ppm-treated samples were not measured due to lack of sufficient tissue (severely damaged plants).

Cellular damage levels were monitored by spectrophotometric determination of lipid peroxidation in leaves of basil plants (**Figure 3A**). Zn 3000 ppm-treated samples were not measured due to lack of sufficient tissue (severely damaged plants). MDA content increases with increasing concentration of heavy metals (Ni, Cu, and Zn) in contaminated soil, except of Ni 500 ppm. No significant differences of lipid peroxidation levels were found in the plants treated with Ni (100, 210, 500 ppm), Cu (200, 500 ppm) and Zn 1500 ppm, compared with control. Moreover, a marked significant increase of MDA content compared with all treatments was verified in the plants treated with Cu 1000 ppm, indicative of maximal cell membrane damage.

### Nitro-Oxidative Responses Following Heavy Metal Application

**FIGURE 4:**
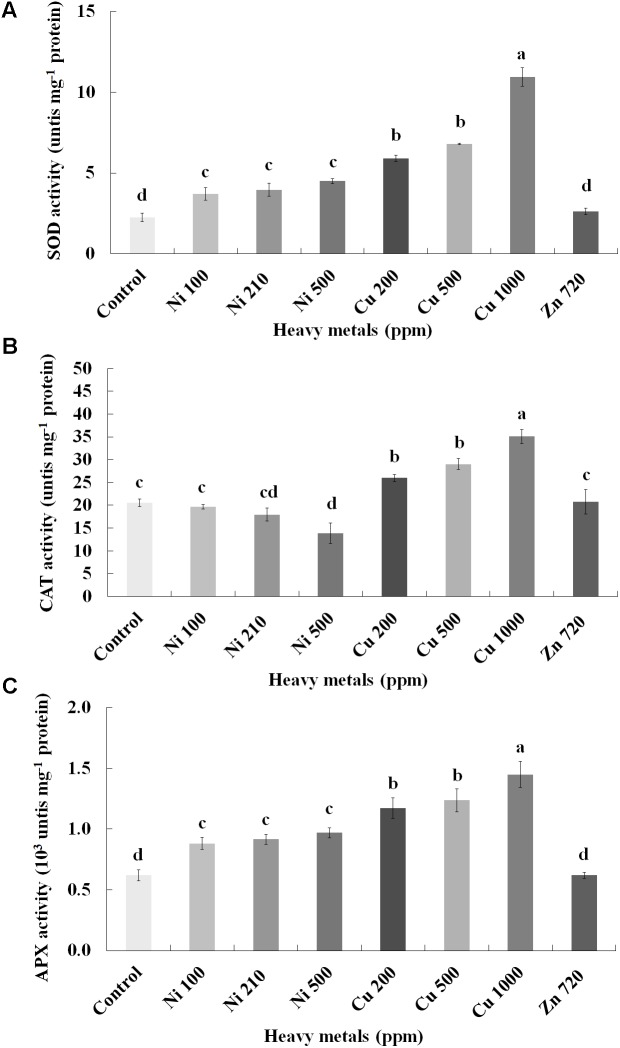
Effects of heavy metals (Ni, Cu, Zn) at three concentrations (including controls), on **(A)** superoxide dismutase (SOD), **(B)** catalase (CAT), and **(C)** ascorbate peroxidase (APX) enzymatic activity of aromatic basil leaves. Data are means ± SE of three replications. Bars with different letters are significantly different at *P* ≤ 0.05. Zn 1500 and 3000 ppm-treated samples were not measured due to lack of sufficient tissue (severely damaged plants).

**FIGURE 5:**
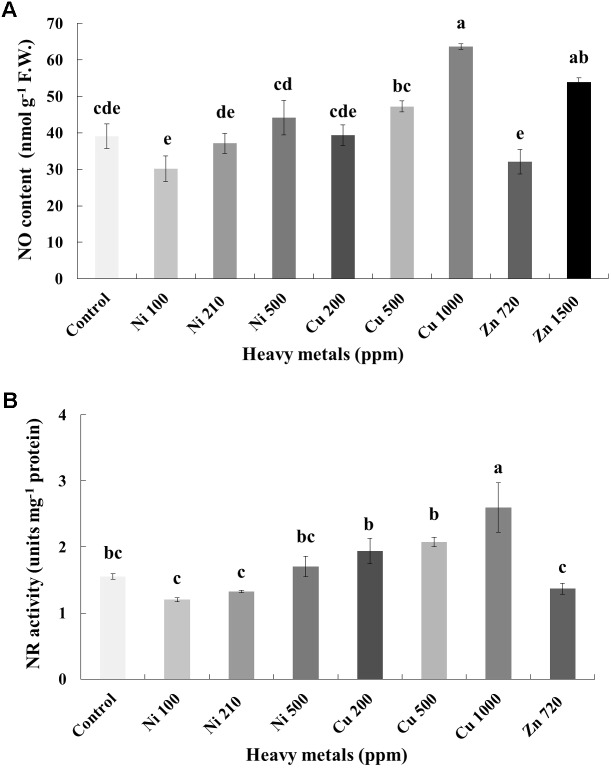
Effects of heavy metals (Ni, Cu, Zn) at three concentrations (including controls), on **(A)** nitrite-derived nitric oxide content (NO), and **(B)** nitrate reductase (NR) enzymatic activity of aromatic basil leaves. Data are means ± SE of three replications. Bars with different letters are significantly different at *P* ≤ 0.05. NO content for Zn 3000 ppm-treated samples and NR activity for Zn 1500 and 3000 ppm-treated samples were not measured due to lack of sufficient amounts of tissue (severely damaged plants).

To investigate if continuous exposure of plants to heavy metals resulted in oxidative or nitrosative stress, the contents of H_2_O_2_ (**Figure 3B**) and NO (**Figure 5A**) were measured in leaves of basil plants. Zn 3000 ppm-treated samples were not measured due to lack of sufficient amount of tissue (severe damage). H_2_O_2_ content increases with increasing concentration of heavy metals (Ni, Cu and Zn) in contaminated soil, except for Cu 500 ppm. Exposure to each of the three heavy metals in three different concentrations resulted in the notable increase of H_2_O_2_ content compared with control, with the increase recorded in plants treated with Cu (200, 500, 1000 ppm) being higher than that in Ni (100, 210, 500 ppm), and Zn 1500 ppm. Notably, plants exposed to the Cu 1000 ppm showed the highest H_2_O_2_ content in leaves compared with all treatments. In turn, plants were found to preserve H_2_O_2_ content similar to control when exposed to Zn 720 ppm (**Figure 3B**). Activity assays of major enzymatic antioxidants SOD, CAT and APX revealed fully correlating patterns with H_2_O_2_ content for all heavy metal treatments with the sole exception of CAT in Ni-treated plants, where enzyme activity remained at control levels and was significantly suppressed following treatment with Ni 500 ppm (**Figure 4**). Zn 1500 ppm and Zn 3000 ppm-treated samples were not measured due to lack of sufficient amount of tissue (severe damage).

In line with H_2_O_2_, NO content increases with increasing concentration of heavy metals (Ni, Cu and Zn) in contaminated soil. No significant differences of NO content were found in the plants treated with Ni (100, 210, 500 ppm), Cu (200, 500 ppm) and Zn 720 ppm, compared with control. Moreover, a marked significant increase of NO content compared with all treatments was verified in the plants treated with Cu 1000 ppm and Zn 1500 ppm, indicative of nitrosative damage (**Figure 5A**). Activity assay of the major NO biosynthetic enzyme in plants NR followed directly correlating patterns with observed NO content, showing significant upregulation in Cu 1000 ppm-treated samples (**Figure 5B**). Zn 1500 ppm and Zn 3000 ppm-treated samples were not measured due to lack of sufficient amount of tissue (severe damage).

### Antioxidant Capacity of Heavy Metal-Treated Basil Plants

To determine the antioxidant capacity in leaves of basil plant of three heavy metals (Ni, Cu, Zn) in three concentrations applied to the soil, phosphomolybdenum and FRAP assays were used (Supplementary Figure S3). Zn 3000 ppm-treated samples were not measured due to insufficient tissue amount resulting from severe damage. In phosphomolybdenum and FRAP, the antioxidant capacity decreases with increasing concentration of heavy metals (Ni, Cu, and Zn) in contaminated soil, with the sole exception of Ni 500 ppm. Phosphomolybdenum and FRAP assays revealed that the experimental treatments (Ni 100 ppm, Ni 210 ppm, and Zn 720 ppm) had no effect on the antioxidant capacity of basil leaves compared with control plants. Phosphomolybdenum assay also showed that Cu 200 ppm did not affect the antioxidant capacity of basil leaves compared with control plants.

### Proline Regulation in Heavy Metal-Treated Basil Plants

**FIGURE 6:**
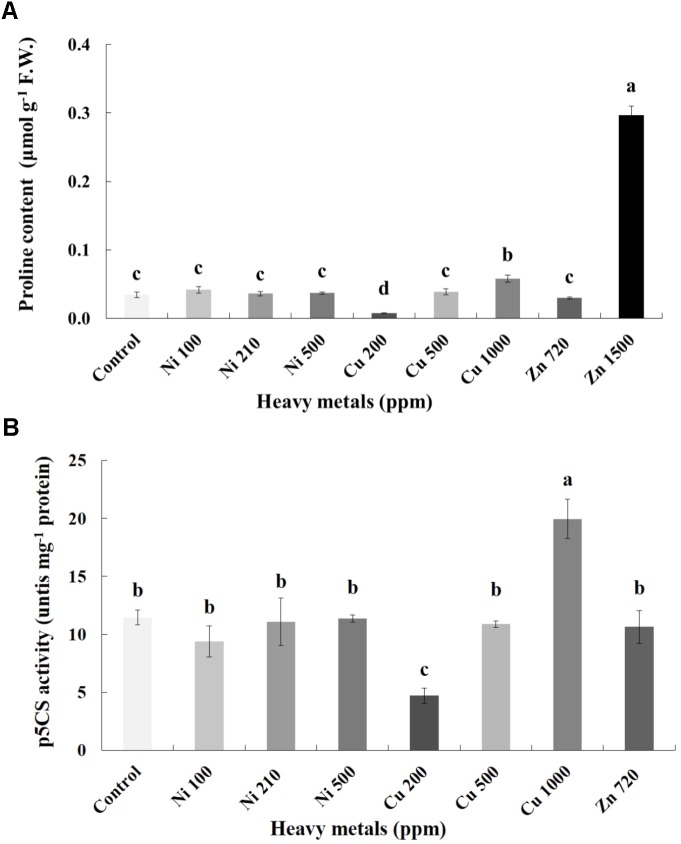
Effects of heavy metals (Ni, Cu, Zn) at three concentrations (including controls), on **(A)** proline content, and **(B)** pyrroline-5-carboxylate synthase (p5CS) enzymatic activity of aromatic basil leaves. Data are means ± SE of three replications. Bars with different letters are significantly different at *P* ≤ 0.05. Proline content for Zn 3000 ppm-treated samples and p5CS activity for Zn 1500 ppm and 3000 ppm-treated samples were not measured due to lack of sufficient amounts of tissue (severely damaged plants).

Proline content was also determined as an additional cellular damage (osmotic stress) indicator. Proline increases with increasing concentration of heavy metals (Cu and Zn) in contaminated soil, except of Ni (100, 210, 500 ppm) in leaves of basil plants (**Figure 6A**). Zn 3000 ppm-treated samples were not measured because there wasn’t enough tissue to perform this analysis. Reduced proline content was recorded in the plants treated with Cu 200 ppm, while it was sustained in levels similar to control in Ni (100, 210, 500 ppm), Cu 500 ppm and Zn 720 ppm. Basil plants grown in the presence of Cu 1000 ppm exhibited higher proline content compared with all Cu treatments. The significantly highest proline content compared with all treatments was Zn 1500 ppm. Activity assay of the major proline biosynthetic enzyme p5CS revealed fully correlating patterns with observed proline content, showing significant suppression in Cu 200 ppm-treated samples, as well as upregulation in Cu 1000 ppm-treated samples (**Figure 6B**). Zn 1500 ppm and Zn 3000 ppm-treated samples were not measured due to lack of sufficient amount of tissue (severe damage).

### Total Protein and Profilin Content of Heavy Metal-Treated Basil Plants

**FIGURE 7:**
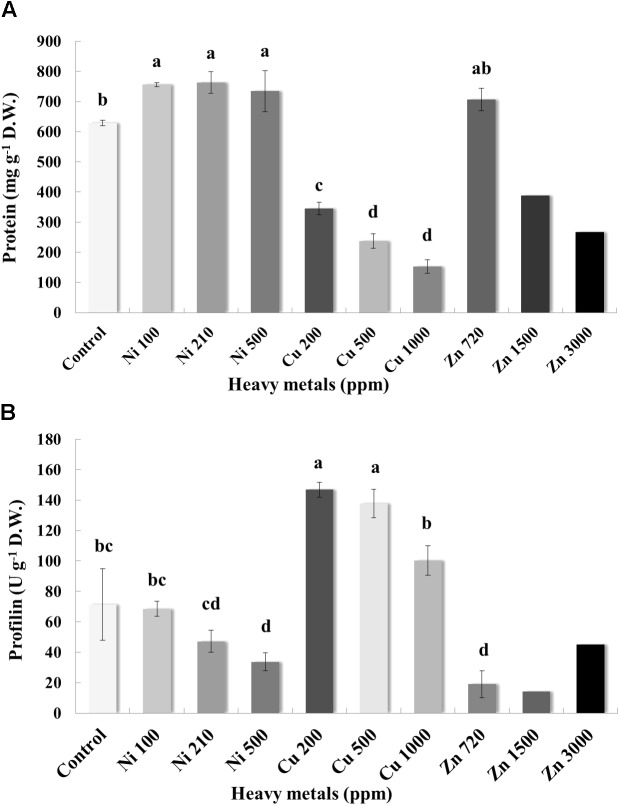
Effects of heavy metals (Ni, Cu, Zn) at three concentrations (including controls), on **(A)** total protein quantity assayed by Pierce Protein Assay, and **(B)** allergenic protein profilin quantity of aromatic basil leaves assayed by ELISA analysis. Data are means ± SE of three replications. Zn 1500 ppm and Zn 3000 ppm-treated values were obtained from one replication. Bars with different letters are significantly different at *P* ≤ 0.05.

Pierce Protein Assay indicated that the concentration of protein in the leaves varies greatly, with control samples containing an average of 628.59 mg g^-1^ D.W. of protein (**Figure 7A**). Plants grown on soil contaminated with Ni were characterized by higher levels of protein when compared with control samples. Basil plants grown on soil contaminated with Cu had a relatively low concentration of protein in the leaves. For Cu contamination of 200 ppm, protein content was 345.15 mg g^-1^ D.W. and almost twice as low as that in control samples. Total protein content decreased with increasing concentration of Cu in the soil (likely due to proteolysis). In the case of Zn-treated plants, total protein content in leaves of basil plants treated with Zn 720 ppm was similar to control samples, whereas protein content decreased significantly with increasing concentrations of Zn in comparison with control samples (again possibly due to proteolysis).

Allergenicity was also assessed by measuring profilin content in control and heavy metal-treated plants. Application of Ni at the lowest and medium concentration (100 and 210 ppm) did not affect profilin content significantly, whereas highest concentration of Ni (500 ppm) resulted in a significant decrease of profilin content compared with controls samples (**Figure 7B**). Interestingly, profilin content in leaves of basil grown in soil contaminated with Cu was significantly higher than that for all other samples, including control ones, with significantly lower profilin content being recorded in plants treated with the highest Cu concentration (1000 ppm) compared with other Cu-treated samples. Finally, plants grown in soil contaminated with Zn at all concentrations (720, 1500, and 3000 ppm) expressed lower profilin content than control samples, with highest profilin content being recorded in plants treated with highest Zn concentration (3000 ppm) among Zn-treated samples.

### Protein Profile and Targeted Protein Identification of Cu 1000 ppm-Treated Basil Plants

**FIGURE 8:**
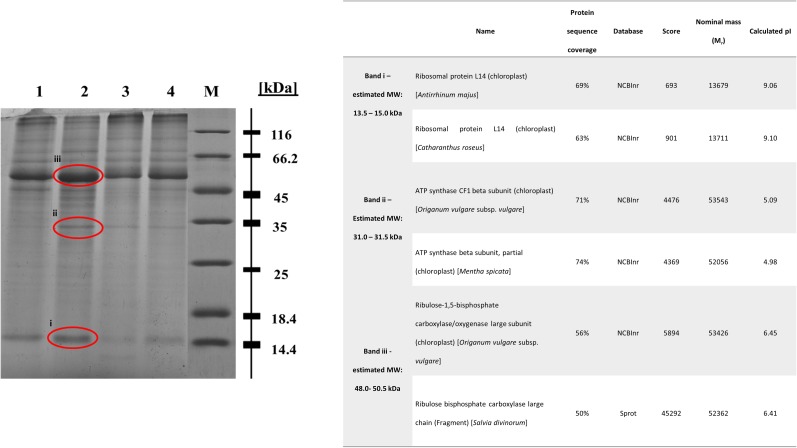
Sodium dodecyl sulfate – polyacrylamide gel electrophoresis (SDS-PAGE) of heavy metal-treated basil plants including control samples (lane 1: control, lane 2: Cu 1000 ppm, lane 3: Ni 500 ppm, lane 4: Zn 720 ppm, M: protein marker). The bands sent for LC-MS-MS/MS method are shown in red circle. Table demonstrates proteins identified in Cu 1000 ppm-treated samples by LC-MS-MS/MS.

In regard with the leaf protein profile of basil plants cultivated in soil contaminated with Cu 1000 ppm, Ni 500 ppm, and Zn 720 ppm compared with control plants, differentially expressed proteins were seen at 13.5–15.0 kDa, 31.0–31.5 kDa, and 48.0–50.5 kDa (**Figure 8**). In basil samples cultivated in soil contaminated with Ni 500 ppm and Zn 720 ppm, protein band density at 13.5–15.0 kDa was lower than in control samples (see Supplementary Table S2), whereas similar MW protein bands were more intense in samples treated with Cu 1000 ppm. Similarly, protein band density at 31.0–31.5 kDa was higher in samples treated with Cu 1000 ppm compared with control ones. The highest protein band densities among samples was observed at 48.0–50.5 kDa MW, with Cu 1000 ppm-treated samples showing highest OD values (Supplementary Table S2).

The differential protein expression profile identified three protein bands showing highest densities in Cu 1000 ppm-treated samples compared with controls and samples treated Ni 500 ppm and Zn 720 ppm (**Figure 8**). These overexpressed bands were subsequently analyzed using liquid chromatography couple to tandem mass spectrometry (LC-MS-MS/MS). Protein band i (**Figure 8**, 13.5–15.0 kDa) was highly homologous to ribosomal protein L14, whereas protein band ii (**Figure 8**, 31.0–31.5 kDa) was homologous to ATP synthase CF1 chloroplastic beta subunit. Finally, protein band iii (**Figure 8**, 48.0–50.5 kDa) showed highest homology to chloroplastic ribulose-1,5-bisphosphate carboxylase/oxygenase large subunit.

## Discussion

Plants exposed to heavy metals produce increased amounts of ROS (Vanhoudt et al., 2010; Yadav, 2010). The overproduction of ROS can affect the redox status of cells which causes dramatic physiological challenges, leading to oxidative stress (Morina et al., 2010). Heavy metal-induced ROS can cause damage to plants, such as enzyme inhibition, protein oxidation, lipid peroxidation (Cuypers et al., 2011), while previous studies showed that ROS production is more toxic on plant macromolecules compared with indirect effects (Pourrut et al., 2011). In general, toxicity is affected by bioavailability of the metals and interactions with other metals in the soil, nutritional status, age and mycorrhizal infection of the plant (Påhlsson, 1989).

Soil uptake of Ni by plants depends on the type and level of soil contamination. Plants absorb large quantities of Ni until they become toxic. The effects of Ni soil contamination on plants takes different forms such as lower seed germination, growth reduction, and decrease of transpiration and photosynthesis (Sreekanth et al., 2013). Cu contributes to the oxidative defense system; however, chronic Cu toxicity can result in severe poisoning (Uriu-Adams and Keen, 2005), as it leads to oxidative damage (Rodriguez-Serrano et al., 2006). Moreover, Zn is a redox-inactive metal which improves the load of ROS but also activates the cellular antioxidant pool and interferes with metabolic balance (Stohs and Bagchi, 1995). Interestingly, a report investigating the contents of nine heavy metals (including Ni, Zn, and Cu) in leaves of eleven types of spices and medicinal plants including basil, revealed that the highest mean levels of Ni and Zn were detected in basil samples, highlighting its increased bioaccumulation capacity (Abou-Arab and Abou Donia, 2000).

Macroscopic plant observations revealed some chlorotic leaves in plants treated with Ni 500 ppm. Plants treated with Cu (200, 500, 1000 ppm) presented similar phenotypes with control plants, as no distinct modulation in plant growth and development was observed, contrary to increased cellular damage levels. Zinc contamination however resulted in highest levels of phenotypic damage, evident through smaller leaves compared with control samples following treatment with Zn 720 ppm, while higher Zn concentrations resulted in severe growth inhibition and chlorosis/necrosis (**Figure 1**). Interestingly, germination rates were only affected negatively in Ni-treated seeds, while Cu and Zn-treated seeds showed similar if not improved germination rates compared with control seeds. The effects of various forms of Ni on seed germination have been studied in the past. According to Leon et al. (2005), Ni chloride (the form used in the present study) was the Ni treatment that resulted in the lowest germination rate compared with other Ni forms (Ni acetate, Ni sulfate) studied, confirming its toxicity on seeds. The differences in the germination rate effects were attributed to Ni salts having specific potential binding sites and properties. Hence, Ni chloride is potentially not as easily absorbed by plant leaves as it is by seeds, resulting in the mild effects on the physiological parameters presented herein. This is further supported by the low concentration of Ni in the leaves, as previously measured by ICP-MS (Szczodrowska et al., 2016).

In regard with photosynthesis-related parameters, pigment content decreased significantly along with increasing concentration of all heavy metals examined (**Figure 2**). A recent report showed that if a plant is exposed to high concentration of Ni, there is a decrease in quantum yield of primary photochemistry and in chlorophyll contents (He et al., 2012). Another report focusing on Cu effects in bamboo plants showed that Cu causes degradation of chlorophyll and that chlorophyll content is reduced considerably when Cu concentration increases from 0 to 2000 ppm Cu (Jiang et al., 2013), while Cu was also shown to inhibit photosynthesis when in high levels (Hou et al., 2007). These results were further supported by the subsequent decrease in physiological processes monitored through SPAD unit measurements in heavy metal-treated basil plants (**Figure 2F**), suggesting that heavy metals can disrupt photosynthetic parameters and photorespiration, changing the normal homeostasis of cells.

Clear indications of cellular damage were observed through the marked increase in MDA content in plants treated with Cu 1000 ppm, indicative of maximal cell membrane damage (**Figure 3A**). Moreover, plants exposed to Cu 1000 ppm also showed the highest H_2_O_2_ content in leaves compared with all other treatments (**Figure 3B**). High amounts of heavy metals (Cu or Fe) contribute to the production of HO^-^ from O_2_^-^ through the Fenton reaction. As a result, the risen level of MDA suggests that metal ions enhance free radical production (Choudhary et al., 2007). Several reports exist supporting this correlation between high levels of Cu linked with increased MDA content (e.g., Hou et al., 2007; Jiang et al., 2013). A further study proposed that Cu enhances free radicals, which in turn can cause non-enzymatic scission of cell-wall polysaccharides resulting in cell-wall loosening (Fry et al., 2002). Nevertheless, highest cellular damage levels manifested though increased MDA and H_2_O_2_ content did not correlate with observed phenotypic damage in the present experimental setup, with the most severely affected samples according to phenotypic observations being those treated with increasing concentrations of Zn. It is possible that the TCA and TBA solutions used in MDA quantification, which contain the electron donors O and S, may sometimes limit Zn thus not allowing similar reaction rates as when it is in ionic form. Furthermore, both MDA and H_2_O_2_ may be reduced because Zn is known to inhibit several cellular enzymatic reactions (Wilson et al., 2012).

In any case, oxidative stress was confirmed to be imposed in all heavy metal-treated samples manifested through increasing amounts of H_2_O_2_, with biochemical evidence for this induction being provided through upregulated activity of major ROS metabolizing enzymes such as SOD, CAT and APX. SOD dismutates superoxide radicals to H_2_O_2_, while CAT and APX act as H_2_O_2_ scavengers (Gill and Tuteja, 2010). Present findings are in line with several reports which demonstrated upregulated enzymatic activity of SOD, CAT and APX following treatment with Cu, Zn and Ni (Choudhary et al., 2007; Mediouni et al., 2009; Duman and Ozturk, 2010), suggesting the occurrence of a ROS scavenging mechanism in an attempt of the plants to defend themselves. Interestingly, a report by Gajewska et al. (2006) examining the effect of Ni in wheat shoots showed a decline in CAT activity following increased Ni amounts, in agreement with present findings (**Figure 4B**). Based on the fact that CAT is an iron-porphyrin and that high concentrations of Ni have been shown to decrease Fe, it was postulated that reduction in CAT activity in plant tissues subjected to excess Ni may result from deficiency of metals essential for biosynthesis of this enzyme molecule (Gajewska et al., 2006). Interestingly, overall antioxidant capacity decreased with increasing concentrations of heavy metals in contaminated soil with the exception of Ni 500 ppm-treated plants (Supplementary Figure S3), which could be the result of the downregulation of other, non-enzymatic antioxidants such as ascorbic acid, glutathione and tocochromanols. Similar results were found in leaves of thyme (*Thymus vulgaris*) plants treated with the same heavy metals as the present report (Kulbat and Leszczyńska, 2016).

Examination of the nitrosative response of plants following heavy metal treatment revealed a similar pattern to that of cellular damage (MDA, proline) levels, in that significantly increased amounts of NO indicative of nitrosative damage were observed in Cu 1000 ppm-treated samples (**Figure 5A**), further supported through an observed upregulation of enzymatic activity of NR, which is thought to be the major biosynthetic enzyme of NO in plants (Mur et al., 2013). Several reports exist which demonstrate the induction of NO biosynthesis under heavy metal treatment (e.g., Yu et al., 2012). During heavy metal stress, different organelles of plant cells can biosynthesize NO in parallel to ROS, such as in mitochondria, chloroplasts. Nitric oxide can alleviate heavy metal-induced ROS, either by stimulating antioxidant defense mechanisms or by directly scavenging (Sahay and Gupta, 2017). Its priming role in specific by acting as a secondary messenger toward the activation of defense mechanisms is attracting increasing attention (Antoniou et al., 2016). Contrarily, excess biosynthesis leads to nitrosative stress and extended cellular damage (Sahay and Gupta, 2017).

Of particular relevance however is a report by Zhang et al. (2008), who demonstrated that increasing concentrations of Cu resulted in concomitant increase in intracellular NO level, correlating with increased proline biosynthesis and upregulated p5CS enzymatic activity in line with present findings (**Figure 6**). The authors proposed that Cu-responsive proline synthesis is closely related to NO generation, suggesting the regulatory function of NO in proline metabolism under heavy metal stress. The proposed model on the nitrosative regulation of proline biosynthesis under heavy metal stress is particularly appealing, as multiple reports exist showing a rise in proline concentration in leaves following Ni, Zn and Cu treatment (e.g., Teklić et al., 2008; Nadgórska-Socha et al., 2013). Higher level of proline increases stress tolerance of plants by employing mechanisms such as osmoregulation, protection of enzymes against denaturation or stabilization of protein synthesis (Sharma and Dietz, 2006; Xu et al., 2009). A possible explanation is the drop in the electron transport system activity under stress which is the reason for accumulation of NaDH and H^+^. The accumulation of proline could be a way to decrease the accumulated NADH and acidity (Venekemp, 1989; Alia, 1991).

Heavy metal contaminants are also known to induce alterations in cellular proteomes. In line with previous reports, treatment with increasing concentrations of Zn and Cu resulted in significant reduction in total protein concentration, possibly due to the degradation of a number of proteins (Duman and Ozturk, 2010). Contrarily, exposure to Ni evoked the highest protein content in all stressed plants when compared with control samples (**Figure 7A**). Such a discrepancy could be related to the higher biosynthesis of specific enzymatic antioxidants, such as SOD following heavy metal contamination (Choudhary et al., 2007), while another study found a similar rise in protein content in the roots and leaves of *N. officinale* up to a certain concentration of Ni (5 mg l^-1^; Duman and Ozturk, 2010). The observed rise may have been the result of the increasing activity of some other metal sequestration mechanisms, found in the detoxification of high Ni doses (Rauser, 1999). Further mass spectrometry analysis of samples treated with high concentrations of heavy metals revealed a significant induction of proteins in basil plants grown in soil contaminated with Cu compared with control samples (**Figure 8**), with upregulated, proteins being identified as ribosomal protein L14, ATP synthase and ribulose-1,5-bisphosphate carboxylase/oxygenase. This came as no surprise as they all represent components of primary metabolism (i.e., respiration, protein synthesis and photosynthesis). Specifically, ATP synthases are membrane-bound enzymes catalyzing the reaction for producing ATP from ADP and inorganic phosphate protein synthesis coupled transport of protons via proton channel across the membrane. The proton gradient is generated by oxidative phosphorylation occurring in mitochondria and photosynthesis in chloroplasts (Boyer, 1997). Ribosomal protein L14 is one of many proteins that form a large ribosomal subunit (60S) and is considered to play a main role in the ribonucleoprotein complex (Davies et al., 1996), while ribulose-1,5-bisphosphate carboxylase/oxygenase (also known as RuBisCO) is a key enzyme involved in the first step of carbon assimilation during the Calvin cycle (Spreitzer and Salvucci, 2002). This observed upregulation comes in agreement with several proteomic analyses of plants under Cu contamination, which identified similar protein components to be induced (Ahsan et al., 2007; Hego et al., 2016; Wang et al., 2017), likely in an attempt by the plants to fuel defense mechanisms in response to stress imposition via increased primary metabolism pathways.

In contrast with the influence of environmental contamination on plant health, allergenicity potential has rather rarely been investigated. It has been previously shown that stimulation of defense mechanisms with salicylic acid or ethylene affects allergenicity levels, as an increase in prohevein (a cross-reactive allergen) in turnip caused by systemic acquired resistance (SAR) activation was claimed (Hänninen et al., 1999). Usually, inhalatory allergens are investigated; however, not much research of influence exists examining environment contamination on allergenicity of consumer (culinary) plants. Cu accumulation leads to the change of cell-walls (Salehi et al., 2014), while increased cell-wall permeability increases protein release due to accelerated diffusion (Aina et al., 2010). In extracts of pollen from plants cultivated in more contaminated areas, allergenic protein synthesis was upregulated (Salehi et al., 2014). As the allergen profile from members of the *Lamiaceae* family has not yet been identified and characterized, it is not known in which way metal contamination in the soil influences their allergenicity. However, there are some reports in the subject of plant allergenicity increase evoked by stress reaction to metals in the soil. For example, exposure of *Poa annua* to Cd in the soil increases their allergenicity, not only because of higher concentration of the major grass pollen allergens but also because of additional allergenic proteins like lipase, nuclease and chitinase-like protein belonging to PR-3 (Aina et al., 2010), although the mechanism of this phenomenon is not clear.

Targeted allergenic examination was carried out by quantification of profilin, an actin-binding protein which plays a role in the dynamics of the cytoskeleton. It is present in the majority of eukaryotic cells (Vidali et al., 2007) and acts as a panallergen, connecting inhalatory allergy and food allergy (Alvarado et al., 2014). These proteins are present in the pollen cells of different plants species, thus rendering them common allergens. Their presence in many plants tissues can explain the connection between pollen sensitivity and food allergies. Profilin takes the important role in the development of food allergy by the person sensitive to grass pollen (Alvarado et al., 2014). In the present study, a differential pattern was observed in that specific concentrations of Ni and Zn resulted in significant decrease in profilin content, whereas Cu contamination – though not at levels damaging to the cell – led to increased profiling levels (**Figure 7B**). The observed rise in profilin content in lower concentration Cu-stressed samples may potentially be linked with increased amounts of salicylic acid in an attempt of the plant to defend itself against the stressor (Mishra and Choudhuri, 1999), and successfully so according to corresponding MDA levels, whereas profilin drops to control levels in severely Cu-stressed samples where damage is significant. Furthermore, an interesting trend was observed in that increasing concentrations of Ni and Cu resulted in a gradual drop in profilin content. This could potentially be linked with chemical properties of the protein, as profilin can bind to groups of proteins which are often on the cytoplasmic side of cell membrane, such as membrane phospholipids and sequences rich in proline. Profilin can be then immobilized leading to polymerization of actin filaments and thus regulating intercellular contacts and cell movement (Mahoney et al., 1999). It is possible that lower profilin content could be caused by smaller extractability of this protein due to increased binding to proline, which is synthesized in response to stressful conditions.

## Conclusion

**FIGURE 9:**
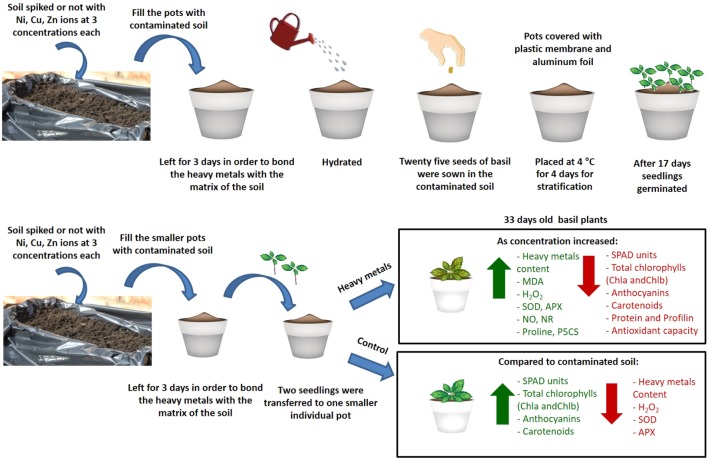
Schematic overview and key results of aromatic basil plants after treatment with three heavy metals (Ni, Cu, Zn) at three concentrations (discussed in detail in the text). Increase is indicated in green arrow and decrease is indicated in red arrow.

The present study provides novel evidence regarding the effects of three heavy metals (Ni, Cu, Zn) applied to the soil on basil plants in three different concentrations compared with control plants (diagrammatic overview of events occurring and key results shown in **Figure 9**). In particular, antioxidant responses and allergen production were investigated. It was found that the highest damage was caused by Cu and Zn. Furthermore, increased concentration of Cu caused a rise in cellular damage as well as in the concentration of profilin, a major allergenic protein, while increased concentrations of Cu and Zn revealed a decrease in the concentration of total proteins (possibly due to proteolysis) and overall antioxidant capacity. Interestingly, concentrations of Ni applied in basil plants decreased allergenic potential and did not appear to be damaging to the plants. Present findings highlight the necessity to further decipher plant responses to heavy metal contaminants due to their toxicity symptoms to plants as well as allergenic potential, potentially employing state-of-the-art systems biology approaches such as gel-free proteomics that will facilitate efforts to improve plant tolerance to these important stressors.

## Author Contributions

JL and VF conceived and designed the experiments. EG, EK, KP, and KK performed the experiments. EG, BS, JL, and VF analyzed the data. EG, JL, and VF wrote the paper. All authors have read and approved the manuscript.

## Conflict of Interest Statement

The authors declare that the research was conducted in the absence of any commercial or financial relationships that could be construed as a potential conflict of interest.
