# Supplementary material for: Influence of Heavy Metals (Ni, Cu, and Zn) on Nitro-Oxidative Stress Responses, Proteome Regulation and Allergen Production in Basil (Ocimum basilicum L.) Plants
Source: Front Plant Sci. 2018 Jul 5;9:862. doi: 10.3389/fpls.2018.00862 (PMC6041727; doi:10.3389/fpls.2018.00862)
Supplement: Supplementary file 1 [file Data_Sheet_1.doc]

**Supplementary material**

**Influence of Heavy Metals (Ni, Cu and Zn) on Nitro-Oxidative Stress Responses, Proteome Regulation and Allergen Production in Basil (*Ocimum basilicum* L.) Plants**

Egli C. Georgiadou^1^, Ewa Kowalska^2^, Katarzyna Patla^2^, Kamila Kulbat^2^, Beata Smolińska^2^, Joanna Leszczyńska^2^ and Vasileios Fotopoulos^1*^

*^1^ Department of Agricultural Science Biotechnology and Food Science, Cyprus University of Technology, Limassol, Cyprus*

*^2^ Institute of General Food Chemistry, Faculty of Biotechnology and Food Sciences, Lodz University of Technology, Łódź, Poland*

*** Correspondence: Vasileios Fotopoulos, email:** [vassilis.fotopoulos@cut.ac.cy](mailto:vassilis.fotopoulos@cut.ac.cy)

**Supplementary Table S1.** Germination rate (%) of control basil plant seeds and seeds treated with heavy metals (Ni, Cu, Zn) at three concentrations.

| **Sample** | **Seeds planted** | **Seeds germinated** | **Germination rate (%)** |
| --- | --- | --- | --- |
| Control | 25 | 18 | 71 |
| Ni 100 ppm | 25 | 13 | 52 |
| Ni 210 ppm | 25 | 16 | 64 |
| Ni 500 ppm | 25 | 16 | 65 |
| Cu 200 ppm | 25 | 21 | 85 |
| Cu 500 ppm | 25 | 18 | 71 |
| Cu 1000 ppm | 25 | 18 | 70 |
| Zn 720 ppm | 25 | 21 | 86 |
| Zn 1500 ppm | 25 | 17 | 67 |
| Zn 3000 ppm | 25 | 19 | 75 |

**Supplementary Table S2.** Protein profile analysis (using KTE Gel Scan) of control and heavy metal-treated basil plants (Cu 1000 ppm, Ni 500 ppm and Zn 720 ppm). Pos (%w), surface area of band (expressed in %); MW, molecular weight; I.O.D., integrated optical density; M.O.D., mean optical density; Area (%), surface area of band.

| **MARKER** | | | | |
| --- | --- | --- | --- | --- |
| **Pos (%w)** | **MW** | **I.O.D.** | **M.O.D.** | **Area (%)** |
| 6.68 | 113.90 | 2583140 | 188 | 6.28 |
| 14.04 | 66.69 | 2333940 | 189 | 5.68 |
| 29.89 | 45.24 | 3974080 | 196 | 9.47 |
| 40.00 | 35.07 | 2507200 | 196 | 5.97 |
| 56.63 | 25.09 | 2672640 | 196 | 6.37 |
| 72.89 | 18.41 | 4183200 | 195 | 9.96 |
| 83.51 | 14.89 | 3898240 | 185 | 9.28 |

| **Control** | | | | | **Cu 1000 ppm** | | | | |
| --- | --- | --- | --- | --- | --- | --- | --- | --- | --- |
| **Pos (%w)** | **MW** | **I.O.D.** | **M.O.D.** | **Area (%)** | **Pos (%w)** | **MW** | **I.O.D.** | **M.O.D.** | **Area (%)** |
|  |  |  |  |  |  |  |  |  |  |
| 2.33 | 156.36 | 1024100 | 169 | 2.50 | 2.03 | 159.77 | 1211140 | 143 | 2.91 |
| 3.69 | 128.17 | 1157280 | 170 | 2.69 | 3.33 | 130.13 | 1063520 | 144 | 2.44 |
| 16.86 | 65.00 | 1122560 | 126 | 2.61 | 16.45 | 65.75 | 1276480 | 135 | 2.93 |
| 20.32 | 59.03 | 1314560 | 137 | 3.06 | 19.86 | 59.79 | 1120000 | 129 | 2.57 |
| 26.65 | 49.50 | 3968160 | 198 | 9.23 | 27.56 | 48.26 | 4447200 | 204 | 10.21 |
| 31.44 | 43.34 | 1983840 | 150 | 4.62 | 31.53 | 43.23 | 1893440 | 148 | 4.35 |
| 34.81 | 39.46 | 931520 | 129 | 2.17 | 34.53 | 39.76 | 1114560 | 142 | 2.56 |
|  |  |  |  |  | 37.18 | 36.94 | 1106720 | 132 | 2.54 |
| 40.23 | 34.91 | 652320 | 122 | 1.52 | 40.23 | 34.91 | 646240 | 124 | 1.48 |
|  |  |  |  |  | 42.19 | 33.56 | 668000 | 124 | 1.53 |
| 45.65 | 31.30 | 714240 | 123 | 1.66 | 45.74 | 31.24 | 1153760 | 142 | 2.65 |
|  |  |  |  |  | 68.20 | 20.22 | 745760 | 118 | 1.71 |
| 88.79 | 13.40 | 2259200 | 142 | 5.26 | 89.38 | 13.24 | 2616160 | 156 | 6.01 |

| **Ni 500 ppm** | | | | | **Zn 720 ppm** | | | | |
| --- | --- | --- | --- | --- | --- | --- | --- | --- | --- |
| **Pos (%w)** | **MW** | **I.O.D.** | **M.O.D.** | **Area (%)** | **Pos (%w)** | **MW** | **I.O.D.** | **M.O.D.** | **Area (%)** |
| 1.56 | 165.27 | 3122560 | 180 | 8.12 | 1.35 | 167.83 | 2999080 | 178 | 7.75 |
|  |  |  |  |  | 4.28 | 125.05 | 1822240 | 157 | 4.48 |
| 11.21 | 93.75 | 737120 | 119 | 1.83 |  |  |  |  |  |
| 15.99 | 66.59 | 1075200 | 117 | 2.68 | 16.04 | 66.50 | 978400 | 123 | 2.41 |
| 19.82 | 59.86 | 1311840 | 126 | 3.27 | 19.73 | 60.02 | 1300960 | 130 | 3.20 |
| 26.06 | 50.32 | 3317600 | 178 | 8.26 | 26.29 | 50.01 | 3685760 | 192 | 9.06 |
| 31.03 | 43.83 | 904320 | 113 | 2.25 | 30.98 | 43.89 | 1350720 | 128 | 3.32 |
| 32.39 | 42.20 | 343200 | 114 | 0.85 |  |  |  |  |  |
| 32.39 | 40.88 | 343200 | 114 | 0.85 |  |  |  |  |  |
|  |  |  |  |  | 33.99 | 40.37 | 1437600 | 118 | 3.54 |
|  |  |  |  |  | 40.32 | 34.85 | 677600 | 110 | 1.67 |
| 45.60 | 31.33 | 810880 | 115 | 2.02 | 45.88 | 31.16 | 854400 | 116 | 2.10 |
|  |  |  |  |  | 89.48 | 13.22 | 2175360 | 128 | 5.35 |


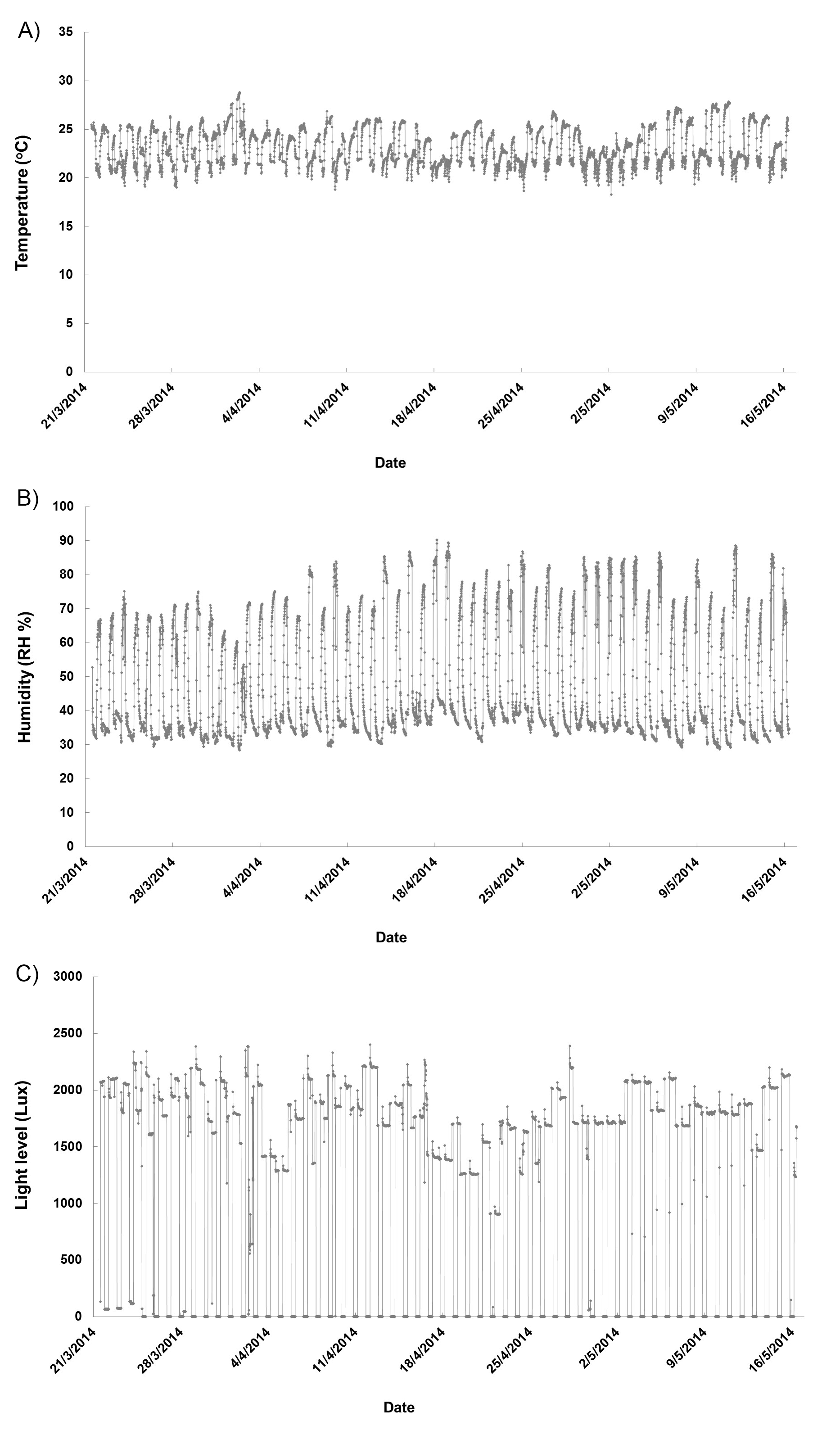


**Supplementary Figure S1.** A) Temperature (^o^ C), B) Humidity (RH %) and C) Light levels (Lux) in the growth room during the growth of basil plants*.*

**
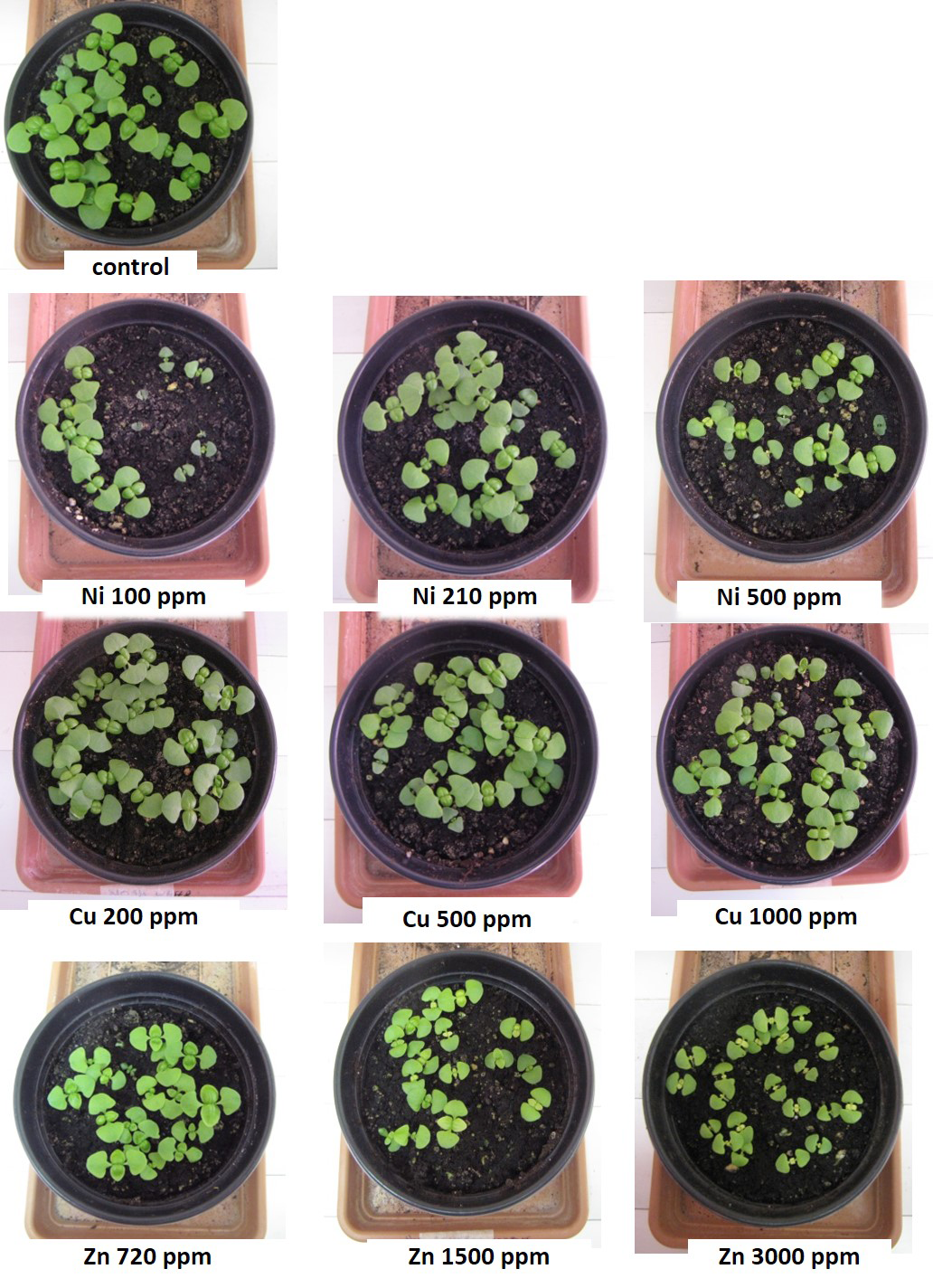
**

**Supplementary Figure S2.** Representation of aromatic basil plants after treatment with three heavy metals (Ni, Cu, Zn) at three concentrations (including control plants). Plants before transplantation (17 d).

**
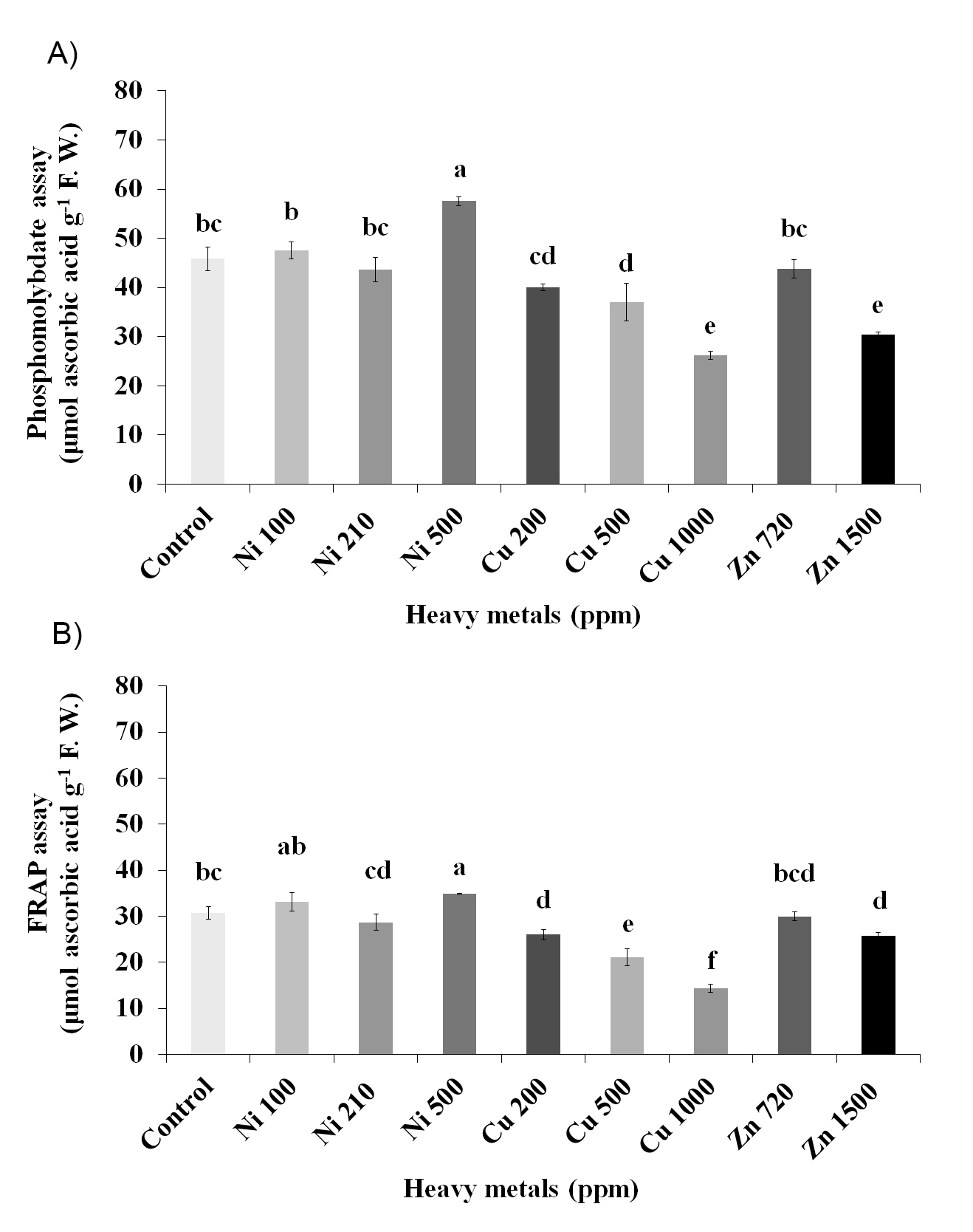
**

**Supplementary Figure S3.** Effects of heavy metals (Ni, Cu, Zn) at three concentrations (including controls), on the antioxidant capacity of aromatic basil leaves measured using A) Phosphomolybdate and B) FRAP assay. Data are means ± SE of three replications. Bars with different letters are significantly different at P ≤ 0.05. Zn 3000 ppm-treated samples were not measured due to insufficient amount of tissue (severe damage).
